# Assessment of salinity tolerance in onion (*Allium cepa* L.) genotypes at germination stage through morphological traits and multivariate analysis

**DOI:** 10.3389/fpls.2025.1686434

**Published:** 2026-01-08

**Authors:** Shreya Kasar, Praveen Roylawar, Bliss Furtado, Kiran Khandagale, Satyabrata Nanda, Snehal Bhandari, Mahendra Khyade, Pranjali Gedam, Hem Raj Bhandari, Vijay Mahajan, Suresh Gawande

**Affiliations:** 1 Department of Botany, S. N. Arts, D. J. Malpani Commerce, and B. N. Sarda Science College, Sangamner, India; 2 ICAR-Directorate of Onion and Garlic Research, Pune, India; 3 Department of Microbiology, Faculty of Biological and Veterinary Science, Nicolaus Copernicus University, Torun, Poland; 4 School of Biotechnology, Centurion University of Technology and Management, Bhubaneswar, Odisha, India

**Keywords:** abiotic stress, germination response, salt injury index, salt stress, sustainable agriculture

## Abstract

Salinity is a major abiotic stress that affects crop growth and development, particularly in crops like onions (*Allium cepa* L.), which are sensitive to saline conditions. Salinity stress limits crop productivity and is difficult to simulate on farms, hindering parental selection for hybridization programs and the development of tolerant cultivars. In this study, 116 A*. cepa* genotypes and released varieties with different genetic backgrounds were used to develop and validate a reliable screening method for salinity tolerance in onion genotypes at early growth stages. The study was conducted in 2 phases. The first was to determine the optimum salt concentration for screening salinity tolerance, and the second was to determine salinity tolerant genotypes and a reliable screening trait for salinity tolerance at the germination/seedling stage. An optimum concentration of 150 mM NaCl, thus found, was used to assess the salinity tolerance of other 100 genotypes by observing key morphological parameters such as germination rate (GR), shoot length (SL), root length (RL), shoot fresh weight (SFW), root fresh weight (RFW), and total fresh weight (TFW). With the help of mean membership function value, and a mathematical model evaluation criteria, the 100 genotypes were categorized into five grades: 6 Highly Salinity Tolerant, 12 Salinity -Tolerant, 59 Moderately Salinity Tolerant, 10 Salinity -Sensitive, and 13 ungerminated genotypes were classified Highly Salinity -Sensitive. Principal Component Analysis was performed to identify key traits contributing to salinity tolerance in *A. cepa* for effective genotype selection. Total fresh weight at 150 mM NaCl was found to be an ideal trait, demonstrating the extent to which *A. cepa* genotypes respond to saline conditions. The methodology within this study presents a simple, efficient, and replicable model for the evaluation of salinity tolerance at germination of onion and other cultivars of economic importance.

## 1 Introduction

Onion (*Allium cepa* L.) is a highly valuable vegetable crop cultivated worldwide, known for its nutritional and medicinal applications. It provides livelihood support to millions of farmers in India, China, Egypt, and many other Asian countries, while the United States, Brazil, and Mexico are major producers outside Asia. The global onion production in 2022 was 75.3 million metric tons (FAOSTAT, 2022); India contributed 28.6% of global production (Directorate General of Commercial Intelligence and StatisticsMinistry of Commerce and Industry, Govt. of India, 2024), making it world’s largest onion producer. India lags behind in per hectare productivity compared to leading countries, and faces considerable challenges like reducing arable land, and increasing costs of cultivation. Abiotic stresses such as heat, drought, and salinity are known to reduce growth and plant productivity in onions (Ghodke et al., 2020; Bose et al., 2025) and other crops by alterations to morphological traits, physiological characteristics, and biochemical and molecular pathways (Suprasanna et al., 2018).

Salinity stress is a major abiotic stress that adversely affects plant growth and productivity, particularly in crops like onions, which are known to be sensitive to saline conditions (Venâncio et al., 2022; Chaudhry et al., 2023). Elevated salt concentrations negatively impact growth parameters including germination, leaf development, and bulb size, ultimately reducing onion yield (Alam et al., 2023). The germination stage is crucial because seedlings are most sensitive to environmental stresses like salinity during this early phase of growth. Previous studies indicate that elevated salinity level significantly impaired germination and reduced onion seedlings vigor (Brakez et al., 2014; Grozeva et al., 2023; Nikolić et al., 2023). The implications of salinity stress extend beyond germination and early growth stages; they can affect the entire life cycle of onion plants. Excessive salinity can lead to significant reductions in crop yield, with biomass losses reported at varying NaCl concentrations (Liu et al., 2022). The physiological mechanisms underlying salinity tolerance in onions involve complex interactions between osmotic stress response, ion homeostasis, and antioxidant defense systems.

A bibliometric analysis of author keyword co-occurrence in Scopus-indexed literature using VOSviewer (Van Eck and Waltman, 2010) revealed 5 clusters of 28 keywords. This highlights strong research focus on antioxidant defense, osmotic regulation, and gene expression under salinity stress (**Supplementary Figure 1**). These findings reflect an interdisciplinary approach integrating molecular biology, physiology, and agronomy.

The genetic diversity among onion genotypes cultivated worldwide provides opportunities for identifying salinity -tolerant lines. This genetic variability can be harnessed through breeding programs aimed at developing cultivars that can adopted to saline environments (Raza et al., 2025). The identification of specific traits linked to salinity tolerance, such as root length, shoot length, and biomass accumulation, can serve as valuable selection criteria in breeding efforts (Sanwal et al., 2022). Understanding physiological, biochemical, and genetic mechanisms of tolerance will enable the development of onion cultivars suitable for cultivation under saline environments (Tarchoun et al., 2022; Areington et al., 2024). Therefore, evaluating onion genotypes for salinity tolerance at the germination stage is not only crucial for immediate seedling establishment but also for ensuring long-term productivity and sustainability in salt-affected agricultural systems. This research is essential for enhancing food security and agricultural sustainability in the face of climate change and soil degradation.

Screening of salinity tolerance levels of plants under field conditions is difficult due to spatial and temporal variability of salt distribution in soil. Such variability reduces reproducibility and hampers reliable genotype screening (Hanci et al., 2016; Aggarwal et al., 2024). *In vitro* studies under controlled conditions overcome these challenges by ensuring uniform stress application for precise and reproducible results. Hence, reliable *in vitro* protocols are crucial for evaluating genotypes for stress tolerance such as salinity stress (Ashraf and Wu, 1994).

Despite numerous studies on salinity tolerance in crops, limited research has focused on identifying seedling-stage responses in diverse onion inbred lines. While previous studies have compared the morpho-physiological and biochemical responses to salinity tolerance in onion, their screening methods were based on limited number of genotypes and whole plants (Sanwal et al., 2022; Solouki et al., 2023; Romo-Pérez et al., 2024). Moreover, these studies did not identify a reliable trait of salinity stress response at germination stage, a pivotal stage of plant life cycle and also nor provided a statistical framework for comparative meta-analysis of large number of genotypes. In this study, 116 A*. cepa* genotypes were used to assess salinity tolerance. This study had two main objectives: (i) to determine the optimum salt concentration for screening, and (ii) to identify a reliable screening trait for salinity tolerance at the germination stage. Additionally, a mathematical model was applied to evaluate salinity tolerance and identify reliable traits. These findings form a foundation for breeding salinity -tolerant *A. cepa* varieties, in line with sustainable and climate-resilient agricultural systems.

## 2 Materials and methods

### 2.1 Plant material

In this study, seeds of 116 onion genotypes and released varieties maintained at ICAR-Directorate of Onion & Garlic Research (DOGR), Pune, India were used (**Supplementary Table 1**).

Experiment design:

The experiment was conducted in September 2022 using *in vitro* germination method in the Plant pathology laboratory, at ICAR-DOGR, Pune, Maharashtra, India, using an *in vitro* germination method. wherein seeds were grown *in vitro* in petri plates (n=15) and amended with the different salt concentrations along with control by maintaining temperature at 23 ± 2 °C, light intensity of 600 μmol m^−2^ s^−1^ (14 h light/10 h dark) and humidity ranging from 50-70%. Triplicates were maintained for each setup.

### 2.2 Determination of optimal salt concentration

A preliminary experiment was conducted using 16 onion genotypes (cultivars and lines) to determine the optimal salt concentration for screening. These lines were subjected to five different NaCl concentrations: 50, 100, 150, 200, and 250 mM, along with a control treatment (distilled water). Before treating the seeds with selected salt concentrations, the seeds of each genotype were surface sterilized by immersion in 70% ethanol for 15 minutes followed by rinsing with sterile distilled water (Aggarwal et al., 2024). The seeds were then imbibed in distilled water for 12 hours, and fifteen uniform and healthy seeds per genotype were selected for further treatment. To conduct the germination study, 90 mm bi-compartment Petri dishes were used, providing an efficient setup for salinity screening. These dishes enabled the simultaneous evaluation of two genotypes under the same treatment conditions, ensuring consistent exposure to salinity stress. Each Petri dish was prepared with a layer of germination paper topped with cotton to create a uniform and supportive growth environment. Seeds from two genotypes subjected to the same NaCl treatment were placed in separate compartments of the dish. Each compartment was then supplied with 10 ml of either distilled water (control) or a designated NaCl solution. To prevent evaporation, Petri dishes were sealed with lids and incubated under controlled conditions with a light intensity of 600 μmol m^−2^ s^−1^ (14 h light/10 h dark). Triplicates were maintained for each setup, and the Petri dishes were monitored regularly, solutions were replenished as needed to maintain salt concentration. Germination was monitored daily for 7 days, and seeds were considered germinated when the radicle length reached ≥ 2 mm. The optimal NaCl stress concentration was determined as the concentration at which the salt-injury index reached 50% compared to the control.

### 2.3 Screening of salinity-tolerant genotypes

The salinity tolerance of 100 A*. cepa* genotypes was evaluated at optimum salt concentration (150 mM NaCl) identified in the preliminary experiment on 16 A*. cepa* genotypes. Before treating with the selected NaCl concentration, a sterilization procedure similar to that used in the preliminary experiment was carried out. Fifteen healthy seeds of each genotype were placed in each half of 90 mm bi-compartment Petri dishes and treated with a 150 mM NaCl solution, while a separate set of control treatments with distilled water was also maintained. The petri dishes were monitored regularly for 7 days, with the solution added as needed. Various morphological parameters, such as the germination percentage, root length, shoot length, fresh weight of roots, and shoot, were measured to evaluate salinity tolerance.

### 2.4 Measurement of morphological traits

The number of germinated seeds was recorded every day for 7 days. The fresh weight and seedling length were also measured at 7 days after sowing (DAS). At the germination stage, morphological traits such as the Germination Rate (GR), Shoot Fresh Weight (SFW), Root Fresh Weight (RFW), Shoot Length (SL) and Root Length (RL) were measured. The germination rate was calculated using the formula below.

Germination rate (GR): Germination rate was calculated 7 DAS:


$$\mathrm{GR}={\text{G}}_{7}/\text{T}\;\times \;100\%$$

Where G_7_ is the number of germinated seeds on 7 DAS, and T is the total number of seeds kept for germination.

SL, RL, SFW, and RFW were measured individually for each replication at 7 DAS. TFW was computed by adding the SFW and RFW. The salinity tolerance index (STI) is the ratio of the NaCl-treated plant’s value to the control’s value. The salt injury index (SII) was calculated by subtracting the STI from 1 (Wu et al., 2019).

### 2.5 Salinity tolerance evaluation

The salinity tolerance of A. cepa was evaluated using the membership function value (MFV) using the fuzzy comprehensive evaluation method (Chen et al., 2012). The MFV of salinity tolerance was calculated using the following equation:


$$\text{Xi}=\frac{\left(\text{X}\;-\;\text{X}\min\right)}{\left(\text{X}\max\;-\;\text{X}\min\right)}\times 100\%$$

Where, Xi - MFV of an i^th^ trait in a specific genotype, X - Actual measured value of STI of that trait in a particular genotype, X_max_ and X_min_ – maximum and minimum STI values observed in all genotypes, respectively (Ding et al., 2018). According to the average value of the MFVs of each trait, the salinity tolerance of the onion genotypes was evaluated. The MFVs of all genotypes ranged from 0 to 1. For each genotype, mean MFV is the average of MFVs of GR, SFW, RFW, SL, RL and TFW. Each genotype had its own mean MFV; higher MFV values indicated greater salt tolerance. According to a previously reported method, the salinity tolerance levels of *A. cepa* were divided into five grades based on the mean of mean MFV values (x̄) and standard deviation (SD) of MFV: (1) Xi≥X ®+1.64SD, Highly SalinitySalt tolerant Tolerant (HST); (2) X ®+1.64SD>Xi≥X ®+1SD, SalinitySalt Tolerant (ST); (3) X ®+1SD>Xi≥X ®-1SD, Moderately Salinity Salt Tolerant (MST); (4) X ®-1SD>Xi≥X ®-1.64 SD, Salinity Salt Sensitive (SS); (5) X ®-1.64 SD≥Xi,, Highly Salinity Sensitive (HSS) (Li et al., 2020; Romo-Pérez et al., 2024) A mathematical evaluation model for salinity tolerance was used:


Y = β1X1+ β2X2 + β3X3 + β4X4 + β5X5 +β6X6+µ

Where Y represents salinity tolerance of *A. cepa* genotypes, X_1_ is the STI of GR, X_2_ is the STI of RL, X_3_ is the STI of SL, X_4_ is the STI of RFW, X_5_ is the SFW and X_6_ is STI of TFW, β is the unstandardized coefficient of relative trait, and µ is constant. Constant (μ) represents the error term.

To validate the initial salinity tolerance classification, a revalidation experiment was conducted. Three genotypes were randomly selected from each salinity -tolerance category, along with all six genotypes from the highly salinity -tolerant (HST) category. The revalidation experiment was performed in triplicates, and each set consisted of 15 seeds per genotype (n=15). The selected genotypes were placed for a second round of screening, following the same experimental methodology as the initial assessment. The validation was based on the comparative evaluation of total fresh weight of germinated seeds. This revalidation ensured the reliability of the classification and confirmed the reliable morphological trait from the observed salinity tolerance responses.

### 2.6 Statistical analysis

Data were analyzed using analysis of variance (ANOVA) to assess the significance of differences among genotypes. Mean comparisons were performed using Tukey’s HSD at a significance level of p< 0.05. The SII, STI, and MFV were calculated manually to evaluate the salinity tolerance of genotypes. Statistical analysis and graphs were prepared using SPSS (version 13.0). Principal Component Analysis (PCA) and Hierarchial Cluster Analysis (HCA) were performed using GRAPES (Gopinath et al., 2021) and SPSS statistical packages, respectively, using normalized data [(x-mean)/standard deviation]. HCA was performed by using parameters that contributed highly to PCA.

## 3 Results

### 3.1 Determination of optimal salt concentration

The optimal salt stress concentration was assessed using 16 A*. cepa* cultivars and genotypes. Data of the morphological parameters such as SL, RL, SFW, RFW, GR and TFW of 16 A*. cepa* genotypes were collected, 7 DAS demonstrating the effect of salt stress on developing seedlings (**Supplementary Table 2**).

**Figure 1:**
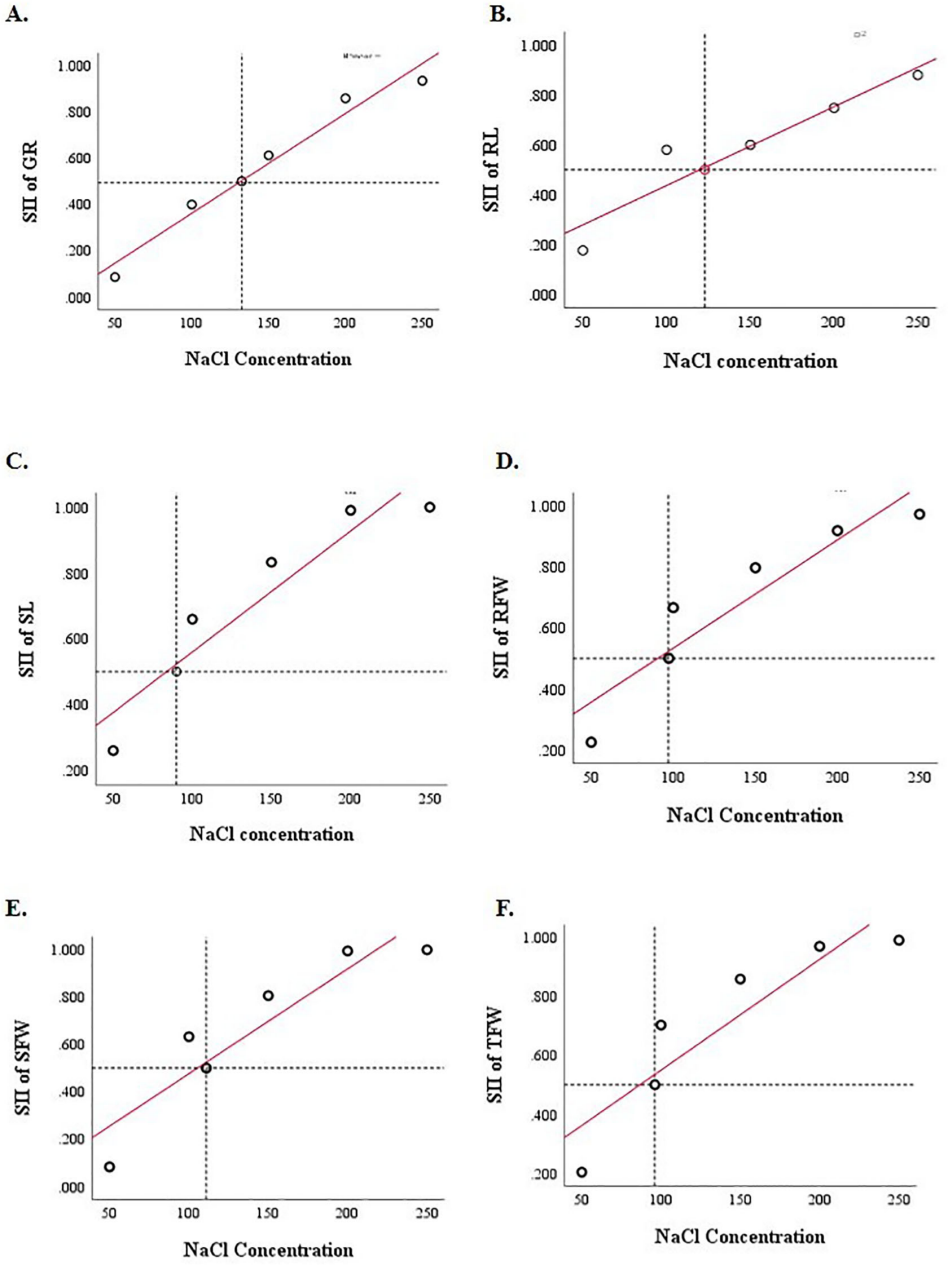
The NaCl concentration of the SII is 0.5 of the GR **(A)**, RL **(B)**, SL **(C)**, RFW **(D)**, SFW **(E)**, TFW **(F)**, of 16 *Allium cepa* varieties under different NaCl concentrations**. Data in the figure are means of 16 *Allium cepa* varieties for each indicator under each concentration of NaCl. **Values are statistically significant at 0.01 level (P< 0.01).

The salt stress concentration was assessed using the SII. It is the threshold at which half of the plants begin to show negative effects from salt stress. In this experiment, when the salt-injury index is 50% as compared to the control, it was selected as the optimal stress concentration of NaCl. Based on the recorded data, the SII of each trait was calculated (**Supplementary Table 3**). The SII of the morphological traits across all 16 A*. cepa* genotypes for each trait was subjected to a linear regression analysis. For GR, 0.5 measure of SII was recorded at 132.5 mM NaCl treatment. Similarly, for SL, RL, SFW, RFW, and TFW the SII of 0.5 was recorded at salt concentrations of 90.1, 122.8, 111.0, 97.2, and 96.1 mM respectively. The 0.5 average SII of all the morphological traits under study was recorded at 110 mM (**Figure 1**). Although an average SII of 0.5 was observed at 110 mM NaCl, indicating moderate inhibition, 150 mM represented the nearest higher concentration in the screening gradient. This level was therefore selected for subsequent evaluations, as it imposed a more pronounced yet sub-lethal stress capable of clearly distinguishing tolerant from sensitive inbreds. Therefore, 150 mM NaCl treatment was used in the present study to evaluate the salinity tolerance of the other 100 onion genotypes.

### 3.2 Correlation analysis of morphological parameters under salt stress

**Figure 2:**
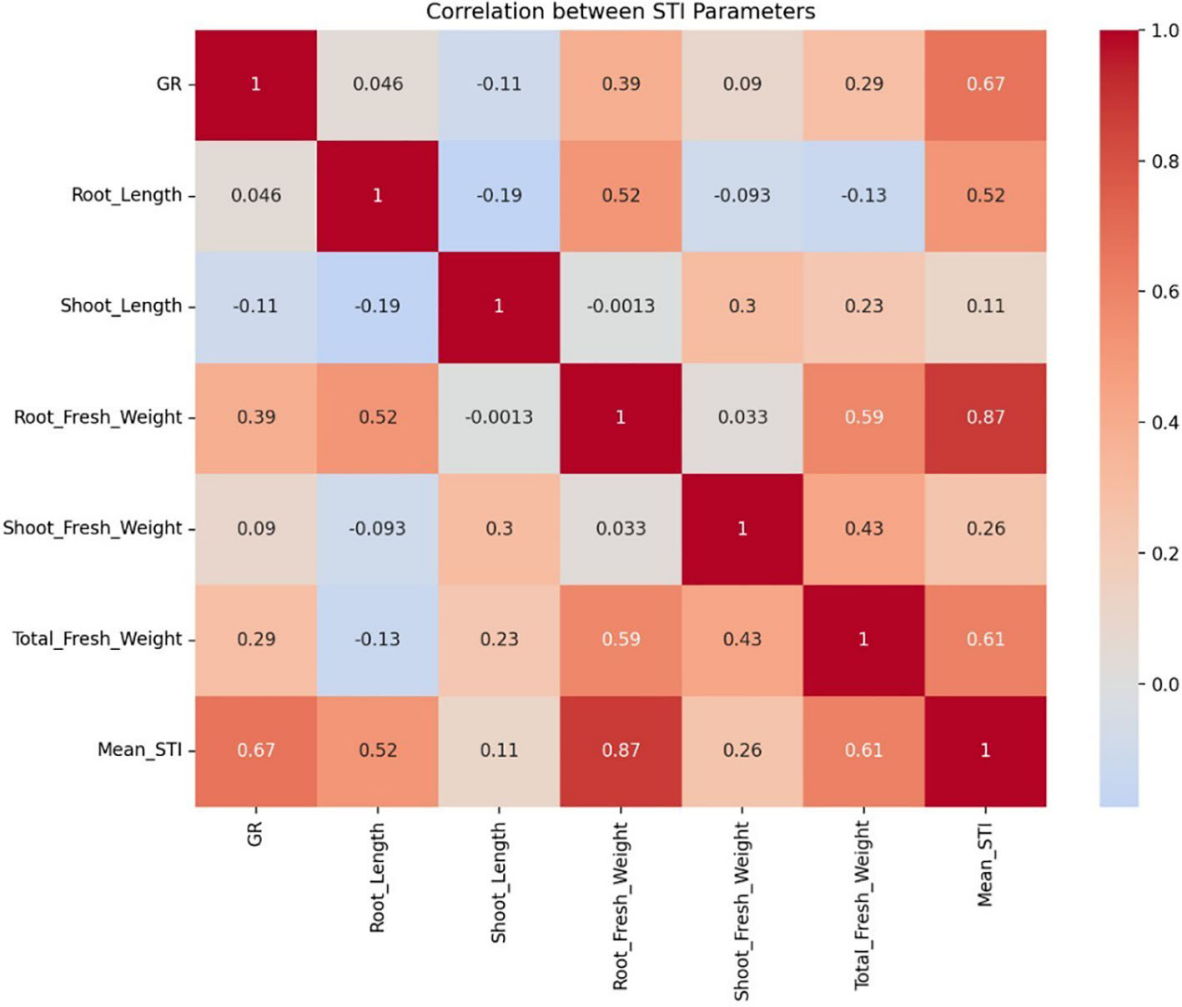
Heatmap of correlation between STIs of different morphological parameters. (Values in each box are p values.).

GR, SFW, RFW, SL, RL, and TFW for each genotype was measured at 150 mM NaCl at 7 DAS, and the STI for each indicator was calculated (**Supplementary Table 4**). To determine if there is any relationship.

between these morphological parameters under NaCl stress, a correlation analysis was performed. The Pearson correlation analysis showed that TFW exhibits the highest positive correlation with RFW (0.59), followed by SFW (0.43). RL showed a moderate positive correlation with RFW (0.52). However, RL displayed weak negative correlations with SFW (-0.09) and TFW (-0.13), suggesting limited contribution under stress conditions. SL had a moderate positive correlation with SFW (0.30) and a weak positive correlation with TFW (0.23). (**Table 1**, **Figure 2**).

**Table 1:** Pearson correlation (r) among salinity tolerance indices (STI) of GR, RL, SL, RFW, SFW, and TFW at 150 mM NaCl (n = 100 genotypes).

Variables (STI)	STI of GR	STI of RL	STI of SL	STI of RFW	STI of SFW	STI of TFW
STI of GR	1	0.046	-0.109	0.388^**^	0.089	0.288^**^
STI of RL	0.046	1	-0.188	0.519^**^	-0.093	-0.133
STI of SL	-0.109	-0.188	1	-0.001	0.303^**^	0.234^*^
STI of RFW	0.388^**^	0.519^**^	-0.001	1	0.033	0.586^**^
STI of SFW	0.089	-0.093	0.303^**^	0.033	1	0.433^**^
STI of TFW	0.288^**^	-0.133	0.234^*^	0.586^**^	0.433^**^	1

*, **Correlation is significant at p< 0.05 and p< 0.01, respectively.

### 3.3 Salinity tolerance evaluation

The MFV of each indicator and the mean MFV were calculated to evaluate the salinity tolerance of *A. cepa* genotypes (**Supplementary Table 5**). The salinity tolerance of 116 A*. cepa* genotypes was divided into five levels: i. HST: Mean MFV ≥ 0.346, ii. ST: 0.346 > Mean MFV ≥ 0.279, iii. MST: 0.279 > Mean MFV ≥ 0.070, iv. Salinity Sensitive SS, 0.070 > Mean MFV ≥ 0.003, v. HSS, 0.003 > Mean MFV.

**Figure 3:**
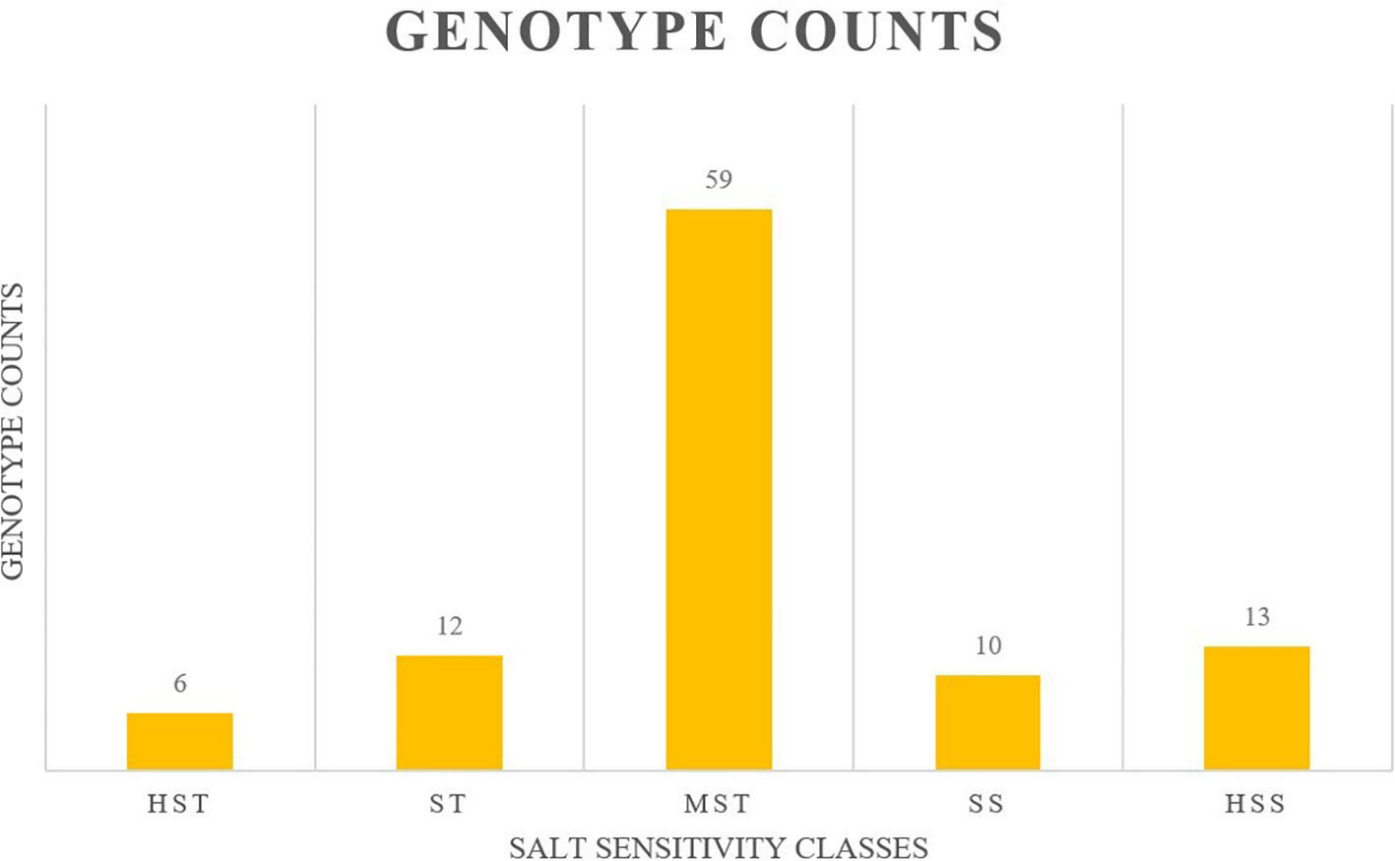
Classification of onion genotypes based on salinity tolerance.

All genotypes were divided into 5 categories, wherein 06 were classified as HST, 12 as ST, 59 as MST, 10 as SS, and 13 as HSS (ungerminated seeds) (**Figures 3**, **Table 2**).

**Table 2:** Classification of 100 A*. cepa* genotypes based on mean MFV values.

HST	ST	MST				SS	HSS
W-210	W-119	W-504	W-444	W-286	W-147	W-009	W-267
W-235	W-394	W-218	W-477	W-187	W-439	W-418	W-043
W-132	W-105	W-329	W-246	W-217	W-221	W-189	W-146
W-331	W-428	W-355	W-215	W-367	W-443	W-225	W-419
W-398	W-160	W-401	W-310	W-208	W-390	W-176	W-226
W-464	W-425	W-275	W-306	W-440	W-397	W-232	W-498
	W-337	W-361	W-515	W-385	W-127	W-081	W-507
	W-396	W-340	W-455	W-174	W-172	W-408	W-151
	W-097	W-414	W-055	W-459	W- 441	W-125	W-138
	W-157	W-405	W-175	W-302	W-277	W-523	W-386
	W-265	W-497	W-087	W-045	W-496		W-182
	W-203	W-177	W-406	W-500	W-353		W-141
		W-344	W-453	W-085	W-442		W-543
		W-431	W-516	W-282	W-364		
		W-255	W-143	W-448			

### 3.4 Salinity tolerance evaluation model and screening for a reliable single indicator

A regression-based evaluation model for analyzing the salinity tolerance of 100 A*. cepa* genotype was established using multiple stepwise regression analysis. The unstandardized coefficients of the STI of GR, SL, RL, RFW, SFW and TFW, were 0.059, 0.059,0.200, 0.075, 0.331, and 0.109 respectively the random error term was -0.01, therefore, Y= -0.01 + 0.059*STI of GR + 0.059*STI of RL + 0.200*STI of SL + 0.075*STI of RFW + 0.331*STI of SFW + 0.109*STI of TFW, where Y represents the salinity tolerance of *A. cepa* genotypes (**Tables 3**, **4**).

**Table 3:** Multiple stepwise regression predicting salt tolerance (Y) from trait STIs at 150 mM NaCl. Include β (unstd), SE, β (standardized), t, p, VIF, model R²/Adj-R²/F/df/p.

Model Summary				
Model	R	R Square	Adjusted R Square	Std. Error of the Estimate
1	.763^a^	0.582	0.577	0.06806
2	.858^b^	0.737	0.730	0.05433
3	.913^c^	0.834	0.828	0.04336
4	.972^d^	0.944	0.941	0.02534
5	.992^e^	0.983	0.982	0.01399
6	1.000^f^	1.000	1.000	0.00031
Model		Unstandardized coefficients		Standardized coefficients	t	Sig.	Lower Bound	Upper Bound	Tolerance	VIF
		B	Std. Error	Beta						
1.	(Constant)	0.094	0.010		9.063	0.000	0.073	0.115		
	TFW	0.243	0.022	0.763	10.873	0.000	0.199	0.288	1.000	1.000
2.	(Constant)	0.046	0.011		4.244	0.000	0.024	0.067		
	TFW	0.260	0.018	0.815	14.433	0.000	0.224	0.296	0.982	1.018
	RL	0.088	0.013	0.397	7.027	0.000	0.063	0.113	0.982	1.018
3.	(Constant)	0.018	0.009		1.926	0.058	-0.001	0.037		
	TFW	0.238	0.015	0.746	16.165	0.000	0.209	0.267	0.937	1.067
	RL	0.100	0.010	0.449	9.828	0.000	0.079	0.120	0.956	1.046
	SL	0.198	0.028	0.326	6.991	0.000	0.142	0.255	0.920	1.087
4.	(Constant)	-0.027	0.007		-4.094	0.000	-0.040	-0.014		
	TFW	0.200	0.009	0.627	21.956	0.000	0.182	0.218	0.836	1.196
	RL	0.095	0.006	0.429	16.022	0.000	0.083	0.107	0.953	1.050
	SL	0.236	0.017	0.388	14.025	0.000	0.203	0.270	0.891	1.122
	GR	0.069	0.005	0.353	12.688	0.000	0.058	0.080	0.881	1.135
5.	(Constant)	-0.028	0.004		-7.794	0.000	-0.036	-0.021		
	TFW	0.173	0.005	0.541	31.859	0.000	0.162	0.183	0.721	1.386
	RL	0.095	0.003	0.429	29.044	0.000	0.089	0.102	0.953	1.050
	SL	0.207	0.010	0.340	21.681	0.000	0.188	0.226	0.847	1.181
	GR	0.069	0.003	0.353	22.941	0.000	0.063	0.075	0.881	1.135
	SFW	0.253	0.018	0.225	13.708	0.000	0.216	0.290	0.769	1.300
6.	(Constant)	-0.010	0.000		-106.959	0.000	-0.010	-0.010		
	TFW	0.109	0.000	0.342	560.351	0.000	0.109	0.110	0.268	3.728
	RL	0.059	0.000	0.264	511.608	0.000	0.058	0.059	0.375	2.664
	SL	0.200	0.000	0.329	953.904	0.000	0.200	0.201	0.842	1.188

a, Predictors, (Constant); TFW, b, Predictors: (Constant), TFW, RL, c, Predictors: (Constant), TFW, RL, SL, d. Predictors: (Constant), TFW, RL, SL, GR, e, Predictors: (Constant), TFW, RL, SL, GR, SFW, f. Predictors: (Constant), TFW, RL, SL, GR, SFW, RFW.

**Table 4:** Agreement between predicted Y and mean MFV for representative genotypes.

Sr. No.	Genotype	Mean MFV	Y	Relative error (%)	Sr. No.	Genotype	Mean MFV	Y	Relative error (%)
1.	W-132	0.456	0.458	0.44	45.	W-397	0.091	0.091	0.00
2.	W-221	0.098	0.099	1.01	46.	W-428	0.317	0.318	0.31
3.	W-147	0.101	0.101	0.00	47.	W-497	0.200	0.200	0.00
4.	W-210	0.507	0.507	0.00	48.	W-516	0.136	0.136	0.00
5.	W-217	0.130	0.130	0.00	49.	W-009	0.069	0.069	0.00
6.	W-310	0.165	0.166	0.60	50.	W-045	0.111	0.111	0.00
7.	W-353	0.076	0.076	0.00	51.	W-055	0.156	0.157	0.64
8.	W-364	0.071	0.071	0.00	52.	W-175	0.143	0.143	0.00
9.	W-405	0.203	0.204	0.49	53.	W-176	0.061	0.061	0.00
10.	W-504	0.246	0.246	0.00	54.	W-189	0.066	0.066	0.00
11.	W-097	0.300	0.301	0.33	55.	W-275	0.212	0.212	0.00
12.	W-119	0.339	0.339	0.00	56.	W-396	0.300	0.300	0.00
13.	W-232	0.049	0.049	0.00	57.	W-442	0.072	0.072	0.00
14.	W-418	0.068	0.068	0.00	58.	W-500	0.111	0.111	0.00
15.	W-464	0.351	0.352	0.28	59.	W-081	0.049	0.049	0.00
16.	W-203	0.284	0.284	0.00	60.	W-331	0.378	0.378	0.00
17.	W-225	0.064	0.064	0.00	61.	W-355	0.231	0.231	0.00
18.	W-265	0.286	0.286	0.00	62.	W-385	0.121	0.120	-0.83
19.	W-286	0.133	0.133	0.00	63.	W-174	0.119	0.119	0.00
20.	W-477	0.173	0.173	0.00	64.	W-177	0.195	0.195	0.00
21.	W-496	0.077	0.077	0.00	65.	W-406	0.138	0.138	0.00
22.	W-408	0.033	0.033	0.00	66.	W-448	0.104	0.104	0.00
23.	W-441	0.085	0.085	0.00	67.	W-453	0.138	0.138	0.00
24.	W-344	0.191	0.191	0.00	68.	W-455	0.158	0.158	0.00
25.	W-515	0.161	0.161	0.00	69.	W-085	0.105	0.105	0.00
26.	W-523	0.020	0.020	0.00	70.	W-087	0.143	0.143	0.00
27.	W-235	0.458	0.458	0.00	71.	W-143	0.134	0.134	0.00
28.	W-282	0.105	0.105	0.00	72.	W-160	0.313	0.313	0.00
29.	W-172	0.086	0.086	0.00	73.	W-208	0.122	0.122	0.00
30.	W-105	0.320	0.320	0.00	74.	W-215	0.167	0.167	0.00
31.	W-157	0.289	0.289	0.00	75.	W-255	0.182	0.182	0.00
32.	W-218	0.243	0.243	0.00	76.	W-306	0.161	0.161	0.00
33.	W-246	0.170	0.170	0.00	77.	W-329	0.238	0.238	0.00
34.	W-337	0.303	0.303	0.00	78.	W-361	0.210	0.210	0.00
35.	W-340	0.207	0.207	0.00	79.	W-367	0.129	0.128	-0.78
36.	W-394	0.325	0.325	0.00	80.	W-390	0.094	0.094	0.00
37.	W-398	0.366	0.366	0.00	81.	W-401	0.215	0.215	0.00
38.	W-414	0.205	0.205	0.00	82.	W-425	0.308	0.308	0.00
39.	W-440	0.121	0.121	0.00	83.	W-431	0.184	0.184	0.00
40.	W-125	0.028	0.028	0.00	84.	W-439	0.100	0.100	0.00
41.	W-127	0.088	0.088	0.00	85.	W-443	0.098	0.098	0.00
42.	W-187	0.131	0.131	0.00	86.	W-444	0.175	0.175	0.00
43.	W-277	0.082	0.082	0.00	87.	W-459	0.117	0.117	0.00
44.	W-302	0.113	0.113	0.00					

To test whether the regression-based evaluation model can predict the salinity tolerance of any genotypes, three genotypes in each of the different clusters were randomly selected and their Y values were calculated. The results indicated that the formula can evaluate the salinity tolerance of any *A. cepa* genotypes at the germination stage. e.g., the Y value of W-210 (HST) is Y= -0.01 + 0.059*1.333 + 0.059*0.500 + 0.200*287 + 0.075*0.704 + 0.331*504 + 0.109*1.208 therefore Y = 0.507, and its mean MFV is 0.507; the Y of W-105 (ST) is 0.320, and its mean MFV is 0.320 and the Y of W-055 (MST) is 0.157, and its mean MFV is 0.156 (**Table 4**).

**Figure 4:**
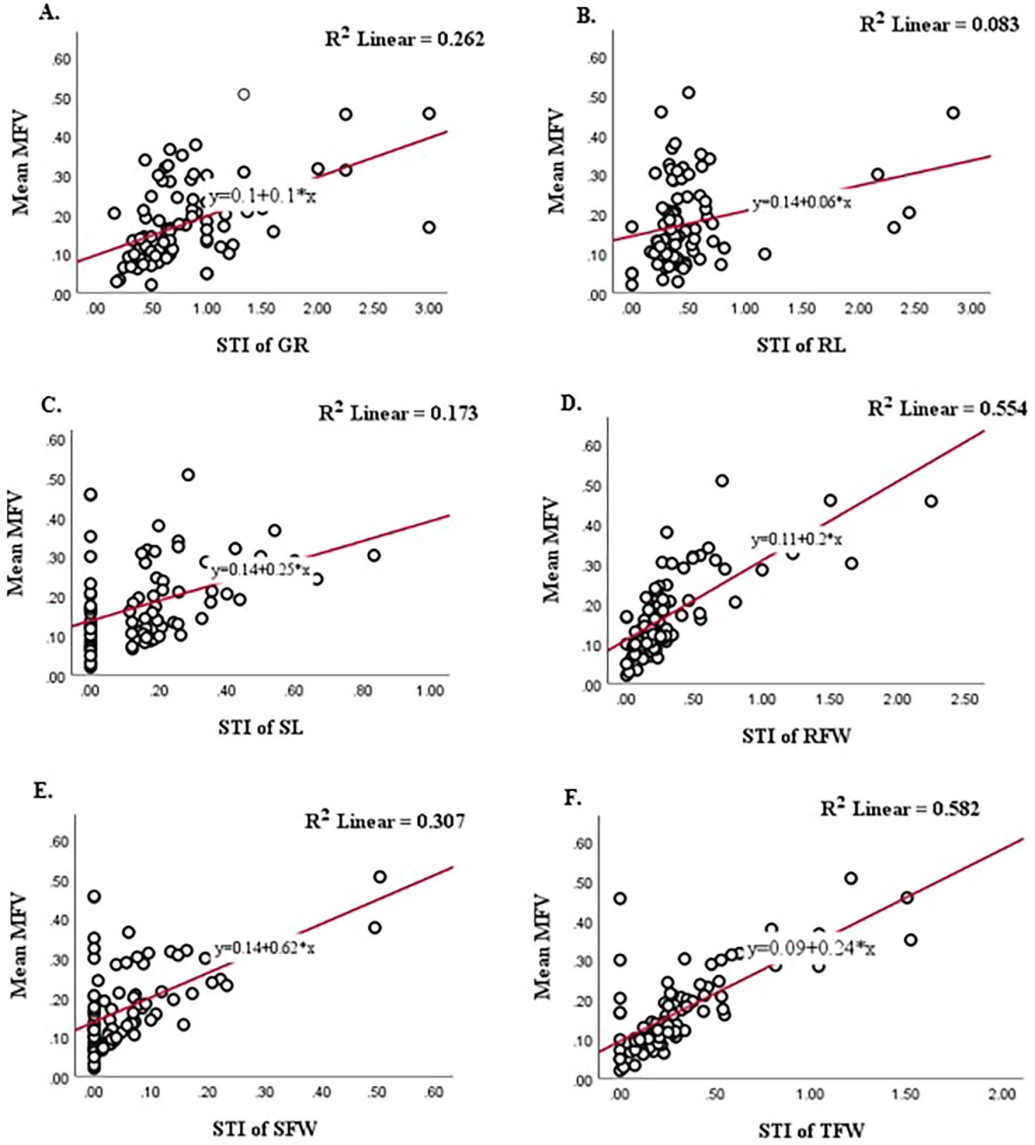
The linear fit between the STI of each indicator and the mean MFV of individual *A. cepa* genotype. **(A)** Is between Mean MFV and STI of GR; **(B)** is between Mean MFV and STI of RL; **(C)** is between Mean MFV and STI of SL; **(D)** is between Mean MFV and STI of RFW; **(E)** is between Mean MFV and STI of SFW; **(F)** is between Mean MFV and STI of TFW.

A higher salinity tolerance (Y value) of the genotypes showed higher growth under mild salt stress as compared to those of controls. Therefore, the model is reliable and salinity tolerance can be predicted by calculating the Y value of any *A. cepa* genotypes using the STIs of growth parameters such as GR, SL, RL, SFW, RFW, and TFW at the germination stage. To determine which indicator is most reliable in reflecting salinity tolerance, a linear model between the STI of each indicator and the mean of MFV was fitted (Li et al., 2020). As shown in **Figure 4**, the R^2^ between the mean MFV and the STI of TFW was the highest (0.582). The R^2^ between the mean MFVs and the STI of RFW, SFW, SL, and GR was comparatively lower, i.e., 0.554, 0.307, 0.173, and 0.262, respectively, and the R^2^ between the mean MFV and the STI of RL was the lowest (0.083). These results are consistent with the results presented in **Table 2**, in which, the standardized beta coefficient between the mean MFV and the STI of TFW was highest. Overall, our results suggest that total fresh weight can be used as a reliable trait to evaluate the salinity tolerance of *A. cepa* genotypes at the germination stage.

**Figure 5:**
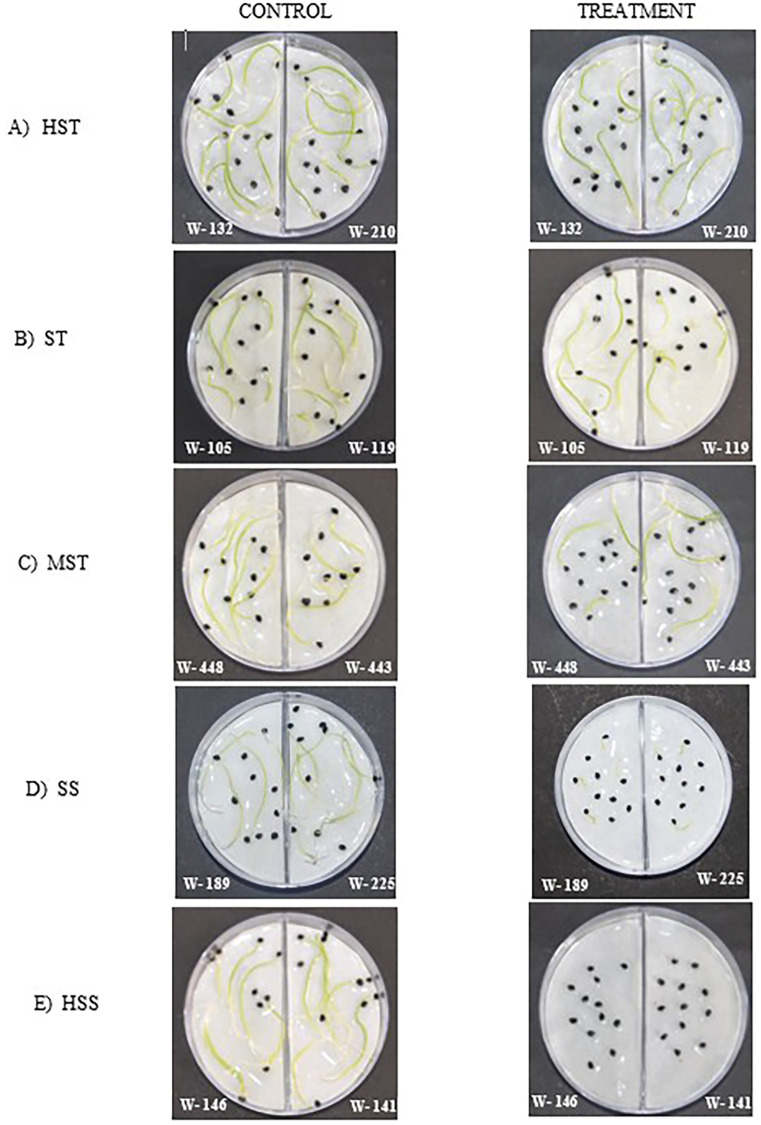
Experimental setup demonstrating phenotypes of the seedlings of **(A)***cepa* genotypes from different salt tolerant categories and their comparison between control (0 mML^-1^) and Treatment (150 mM NaCl) at 7 DAS. **(A)** HST, **(B)** ST, **(C)** MST, **(D)** SS, **(E)** HSS.

To verify the obtained results, three genotypes were randomly selected from each salt-resistant category along with all 6 genotypes of the HST category, and the TFW was determined at 7 DAS (n=15) in 150 mM NaCl. No significant difference (P > 0.05) in growth existed between the different *A. cepa* genotypes in the control. However, the growth and Total fresh weight were significantly reduced by NaCl as the salinity tolerance of the *A. cepa* genotypes decreased (HST > ST> MST > SS > HSS). The HSS *A. cepa* genotypes barely germinated in 150 mM NaClNaCl while all the HST genotypes showed germination under salt stress of 150 mM NaCl. The average TFW of an individual plant was 54 mg (HST lines), 20 mg (ST lines), 14 mg (MST lines), and 10 mg (SS lines). Furthermore, TFW of HSS *A. cepa* lines was close to 0. (**Figure 5**).

### 3.5 Principal component analysis

PCA was employed to explore the variability among six morphological parameters across genotypes and identify key traits contributing to the observed variance. The first two principal components (PC1 and PC2) accounted for 60.97% of the total variance, with PC1 explaining 33.94% and PC2 contributing 27.03%. PC1 was primarily influenced by TFW and RFW, which contributed 34.42% and 33.48% to this component, respectively. PC2 was dominated by RL and SL, with contributions of 32.68% and 26.48%, respectively.

**Figure 6:**
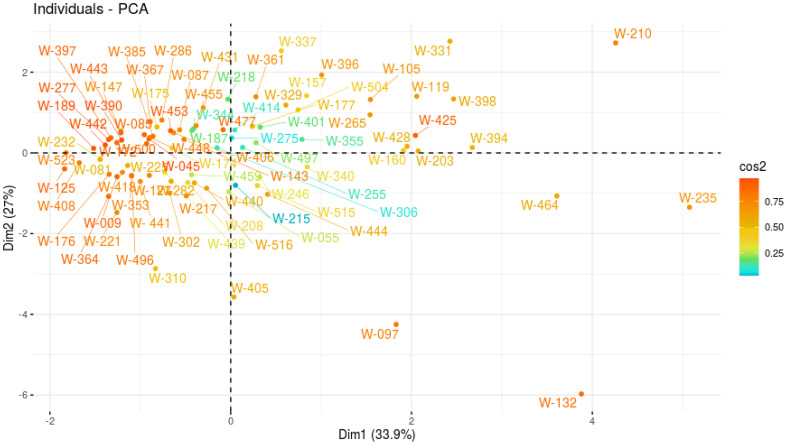
PCA of STI of 100 A*. cepa* genotypes under 150 mM salt concentration.

**Figure 7:**
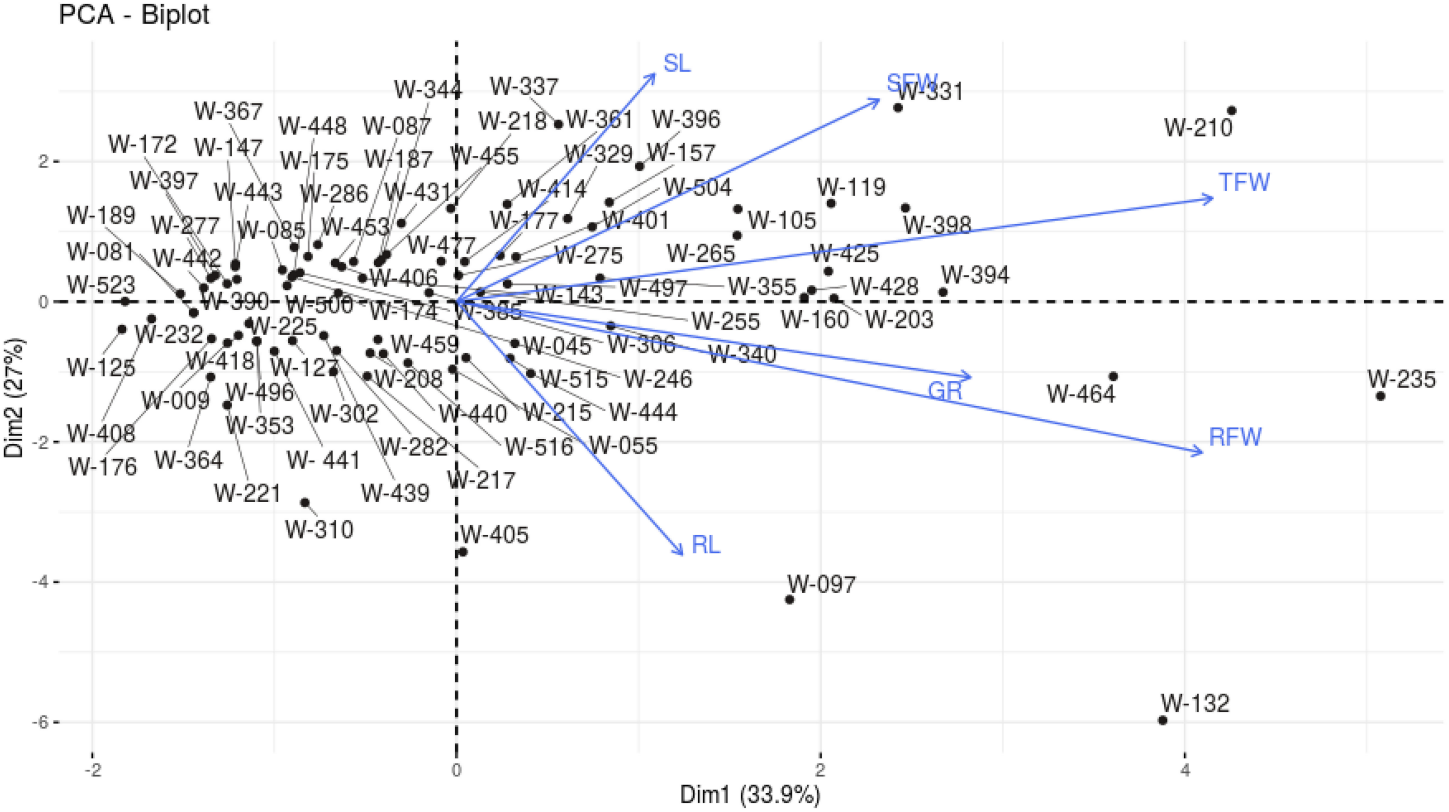
PCA Biplot of 100 A*. cepa* genotypes based on STI of 6 morphological parameters under 150 mM NaCl.

The PCA biplot (**Figures 6**, **7**) represents genotypic clustering and trait contributions. Genotypes such as W-210 and W-235 exhibited higher TFW and RFW values, aligning positively with PC1. In contrast, genotypes like W-097 and W-132 were distinguished by traits related to RL and SL, projecting along PC2. The arrows representing traits in the biplot demonstrate the strength and direction of their contribution to the principal components, with longer arrows indicating stronger contributions.

### 3.6 Hierarchical cluster analysis

**Figure 8:**
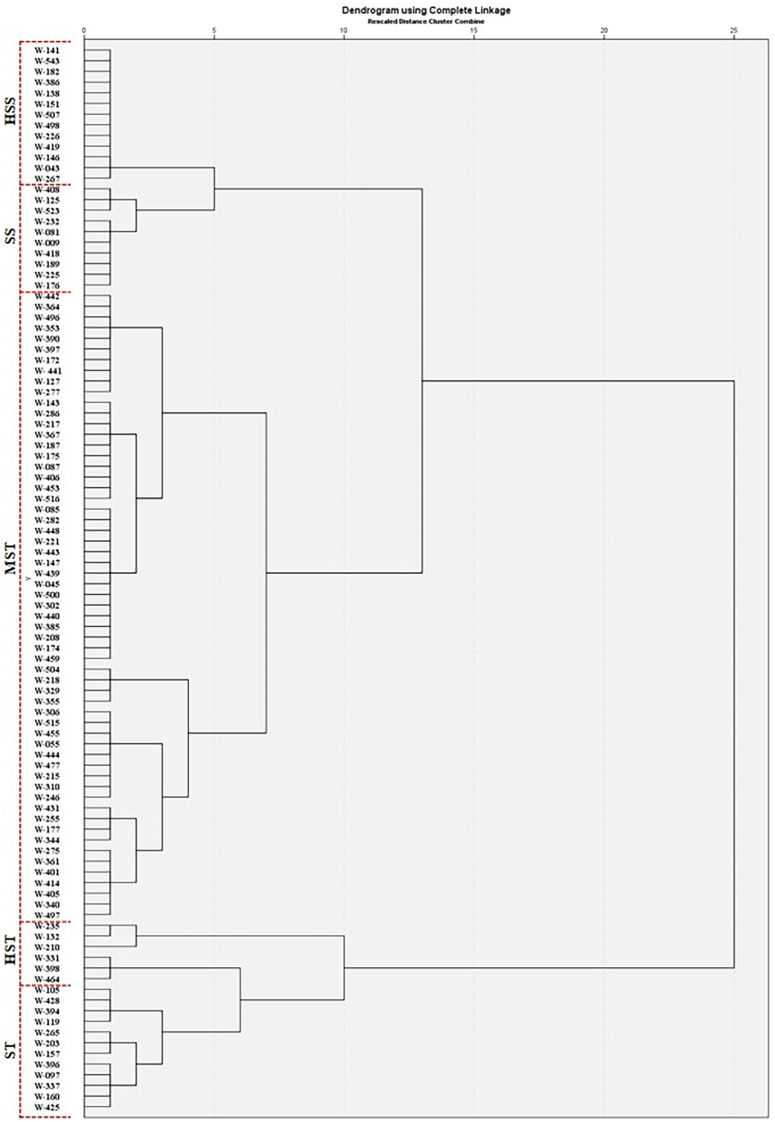
HCA based on the Furthest Neighbor to evaluate the salt tolerance of 100 A*. cepa* genotypes.

The MFV and mean MFV of each indicator were used to perform HCA and classification of 100 A*. cepa* genotypes. A higher mean MFV indicates stronger salinity tolerance. A hierarchical cluster analysis based on the Ward’s linkage was utilized to classify 100 genotypes into five categories based on their mean MFV values (**Figure 8**).

## 4 Discussion

Salinity is a major abiotic stress that can severely impact plant growth and productivity, affecting nearly ~20% of the world’s cultivated land (Nikalje et al., 2018). The genus *Allium* (Family: Amaryllidaceae) is an economically important group of crops cultivated worldwide for their use as a vegetable and spices (Khandagale et al., 2020). However, its cultivation faces several challenges, particularly in arid and semi-arid regions where salinity poses a significant constraint. This underscores the importance of evaluating crops and developing the genotypes that can thrive under such conditions (Mangal et al., 1989). Salinity stress affects plant growth and development by causing osmotic stress, ion toxicity, nutrient imbalances, and oxidative damage, which collectively impair germination, seedling growth, and overall plant vigor (Bandehagh and Taylor, 2020). Plants are generally extremely sensitive to soil salinity during germination and early growth (Çavuşoğlu, 2023). Thus screening for salinity -tolerant genotypes is essential to identify plants capable of maintaining growth and productivity under saline conditions. By selecting salinity -tolerant genotypes, breeding programs can develop resilient varieties suited for cultivation in salinity -affected soils, ensuring sustainable agricultural production and food security (Ashraf and Wu, 1994; Atta et al., 2023). A study of effect of salinity on amaranth genotypes found higher accumulation of Na^+^ and corresponding lower accumulation of K^+^ in root and shoot regions of seedlings (Tebini et al., 2022). Ionic accumulation has been reported to cause decrease in enzymic conversion of reserve food material in lentil embryos (Foti et al., 2019) and also disrupt Na^+^/K^+^ homeostasis, causing hyperosmosis in rapeseed cultivars (El-Badri et al., 2021). In onion, salinity stress triggered a biphasic tolerance response at 50 mM NaCl and higher. Fresh weight (bulb, root, leaves) and relative water content decreased, while osmotic potential, transpiration, and photosynthetic pigments increased. Leaf, root, and bulb K^+^ and Ca^+^ declined, whereas Na^+^ and Zn increased. Aquaporin genes PIP1, PIP2, and TIP2 were generally downregulated in roots but remained stable in leaves and bulbs (Solouki et al., 2023). A previous study by Sanwal et al., 2022 compared 36 accessions of onion for salinity tolerance in terms of physio-biochemical traits and antioxidant enzymes and reported 14-63% bulb yield reduction, 28-39% lower photosynthetic rate, 9-96% greater proline content, 24-149% increase in activity of antioxidant enzymes, catalase, ascorbate peroxidase, superoxide dismutase and peroxidase (Sanwal et al., 2022). A study focusing on metabolic response to salinity stress in onion found reduced leaf levels of succinate, fumaric acid, 2,3-dihydroxybutyric acid, 2,4-dihydroxybutyric acid, malic acid, ribonic acid and erythronic acid (Romo-Pérez et al., 2024).

Various studies have explored salinity tolerance in different crop plants including brassica (Wu et al., 2019; Dai et al., 2024; Aggarwal et al., 2024), oats (Bai et al., 2018), upland cotton (Sikder et al., 2020), Helianthus (Li et al., 2020) etc. at seed germination and early seedling stage in controlled conditions. In ajwain (*Trachyspermum ammi* [L.] Sprague), due to Na+ and Cl- accumulation, K^+^/Na^+^ and Ca^2+^/Na^+^ ratios in the shoots were higher in comparison to roots (Ashraf and Orooj, 2006).

In the present study, a total of 116 A*. cepa* genotypes and released varieties were screened to study their salt tolerance at the germination stage. Since the germination rate alone cannot capture the complex physiological responses to salinity, it is insufficient for accurately evaluating salinity tolerance. Therefore, we incorporated multiple morphological traits, such as shoot and root length, and fresh weight of shoot and root, along with the germination rate, to provide a more comprehensive assessment of salinity tolerance in *A. cepa* genotypes. Initially, five different salt concentrations were used to screen 16 genotypes of *A. cepa* at the germination and seedling stage to determine a single effective salt concentration for screening purposes. For this study, we utilized bi-compartment Petri dishes. This allowed the simultaneous cultivation of two genotypes under identical controlled conditions, facilitating direct comparison and ensuring consistency in experimental parameters. Different concentrations were observed to affect germination rate, fresh weight, root, and shoot length in different ways. As compared to control, higher salt concentrations tend to significantly decrease the values of physiological and morphological traits. In this study, the SII and STI were employed to comprehensively evaluate the response of *Allium* genotypes to salinity stress. Utilizing either the SII or STI has proven advantageous for investigating salt stress at both the phenotypic and molecular levels. Preliminary screening was performed using a gradient of NaCl concentrations to assess the response of inbred lines under varying salinity levels. Although an average SII of 0.5 was observed at 110 mM NaCl, indicating moderate inhibition, 150 mM represented the nearest higher concentration in the screening gradient. This level was therefore selected for subsequent evaluations, as it imposed a more pronounced yet sub-lethal stress capable of clearly distinguishing tolerant from sensitive inbreds. Subsequently, tolerance of all the other 100 genotypes was determined under 150 mM NaCl concentration for the same parameters and analyzed using the mean MFV to assess salinity tolerance across multiple traits. MFV is an important indicator for evaluating salinity tolerance as it facilitated the ranking of genotypes based on their overall salinity tolerance by considering multiple traits simultaneously thus higher the mean MFV value, the higher the salinity tolerance.

All genotypes were divided into 5 categories, where, 06 genotypes were classified as HST, 12 genotypes as ST, 59 genotypes as MST, 10 genotypes as SS, and 13 ungerminated genotypes were considered as HSS (**Figure 4**). Some of the genotypes exhibited lower mean MFV, indicating they have high salt sensitivity at the germination stage. In our study, the mean MFV was affected by the STI of GR, SL, RL, SFW, RFW, and TFW, which means that the higher the STI value of each indicator, the higher the MFV value. Greater suppression of root and shoot growth in sensitive classes is consistent with osmotic/ionic stress and ROS burden; tolerant lines likely maintain better ion homeostasis and antioxidant capacity (Ding et al., 2018; Kesawat et al., 2023). A mathematical formula based on the multiple stepwise regression analysis was used for the reliable and efficient evaluation of salinity tolerance in *A. cepa* genotypes. The mathematical model proved to be the time-saving and convenient model for screening salinity tolerance. The Y value was estimated for all the genotypes (**Supplementary Table S6**) and used for the accurate evaluation of salinity tolerance using the model. The highest value of Y depicted the higher salinity tolerance. which is in accordance with the results obtained by MFV.

Correlation analysis and linear fit model were used to find the relationship between various morphological parameters under NaCl stress based on the STI values of GR, RL, SL, RFW, SFW and TFW. According to the results of the correlation analysis, the STI of TFW exhibits the highest positive correlation with Root Fresh Weight (RFW) (0.59), followed by SFW (0.43). This highlights the significant contribution of root and shoot biomass to total biomass under salt stress. RL showed a moderate positive correlation with RFW (0.52), indicating that longer roots promote higher root biomass. SL had a moderate positive correlation with SFW (0.3) and a weak positive correlation with TFW (0.23), suggesting its minor role in biomass accumulation. GR showed a moderate correlation with Mean STI (0.67) and TFW (0.29), indicating a potential indirect effect on salinity tolerance. The linear fit model showed that the STI of TFW had a high coefficient of determination with mean MFV (R^2^ = 0.582). Thus, TFW can be used as a reliable trait for screening salinity-tolerant *A. cepa* genotypes on a large scale during the germination stage.

The revalidation experiment confirmed the initial classification of *A. cepa* genotypes based on salinity tolerance. In the control treatment, no significant differences (P > 0.05) in growth were observed among the genotypes, indicating uniform growth potential in the absence of salt stress. However, under 150 mM NaCl stress, significant reductions in growth and total fresh weight were observed, with the extent of reduction correlating with the salinity tolerance level of each category. HSS genotypes exhibited minimal to no germination at 150 mM NaCl, whereas all HST genotypes successfully germinated under these conditions. TFW of individual plants followed the expected trend, with HST lines maintaining the highest fresh weight (54 mg per plant), followed by ST (20 mg), MST (14 mg), and SS (10 mg) lines. HSS lines displayed a near-zero total fresh weight, further confirming their extreme sensitivity to salinity. These findings support the results of the initial classification and demonstrate the strong correlation between salinity tolerance and total fresh weight under saline conditions. The consistent trend observed across both experiments highlights the reliability of the screening methodology in distinguishing salinity-tolerant and salinity-sensitive *A. cepa* genotypes.

PCA was utilized to analyze the variability among genotypes based on six morphological parameters. PC1 was predominantly influenced by TFW and RFW, with high positive loadings, indicating their significance in distinguishing the genotypes. Conversely, PC2 was largely associated with RL and SL, reflecting their independent contribution to variability. This indicates that fresh weight parameters play a major role in explaining overall variability, while length parameters exhibit a distinct and independent influence. Furthermore, genotypes with higher scores on PC1 were characterized by greater fresh weight, whereas those scoring higher on PC2 displayed variations in length parameters. The strong correlation of TFW (0.837) and RFW (0.826) with PC1, and RL (-0.728) and SL (0.655) with PC2, underscores the significance of these traits in distinguishing genotypic performance. These findings suggest that targeting fresh weight and length traits could be pivotal in selecting genotypes with superior morphological performance.

The findings of this study on salinity screening in *Allium* genotypes align with similar research conducted on other crops such as Sunflower and Brassica (Li et al., 2020; Aggarwal et al., 2024), though notable differences were observed due to crop-specific responses to salinity. For instance, the findings of this study highlight TFW as a reliable trait to determine salinity tolerance, whereas in *Brassica napus* SFW can be considered as a reliable trait for salinity tolerance screening of genotypes (Aggarwal et al., 2024). Similarly, studies reported the germination Index as a reliable trait to analyze the salinity tolerance of sunflower genotypes (Li et al., 2020). These variations emphasize the species-dependent nature of salinity tolerance and the importance of considering multiple morphological traits in screening studies.

Future studies will aim to validate these preliminary findings under pot and field conditions with natural soil salinity gradients. Additional validation through physiological markers, biochemical assays, and gene expression analysis is also planned to support the screening outcomes at a molecular level.

## 5 Conclusions

Onion is an important vegetable crop cultivated worldwide and is susceptible to a variety of abiotic stresses including salinity stress, which causes reduction in seed germination, disrupted ion homeostasis and reduction in bulb yield. The optimal salt concentration of 150 mM NaCl was determined for salinity tolerant evaluation of 100 A*. cepa* genotypes. Furthermore a regression-based model was used for the evaluation and classification of salinity tolerance of *A. cepa* and classified in to 5 categories as HST (n = 6), ST (n = 12), MST (n = 59), SS (n = 10), and HSS (n = 13). These findings suggest that RFW and TFW are key predictors of salinity tolerance, making them valuable traits for breeding programs focused on improving salinity resilience in onion genotypes. To assess salinity stress response at seed germination and compare the performance of different genotypes for multiple other crops, the screening method used in this study can be used as a basis. While the present work provides a rapid screening method under lab conditions, further validation in field environments and integration with physiological and molecular data will be essential to confirm and utilize these genotypes in breeding programs.

## Data Availability

The original contributions presented in the study are included in the article/**Supplementary Material**. Further inquiries can be directed to the corresponding author.
