# Supplementary figures and images for: Assessment of salinity tolerance in onion (Allium cepa L.) genotypes at germination stage through morphological traits and multivariate analysis
Source: Front Plant Sci. 2026 Jan 8;16:1686434. doi: 10.3389/fpls.2025.1686434 (PMC12823497; doi:10.3389/fpls.2025.1686434)

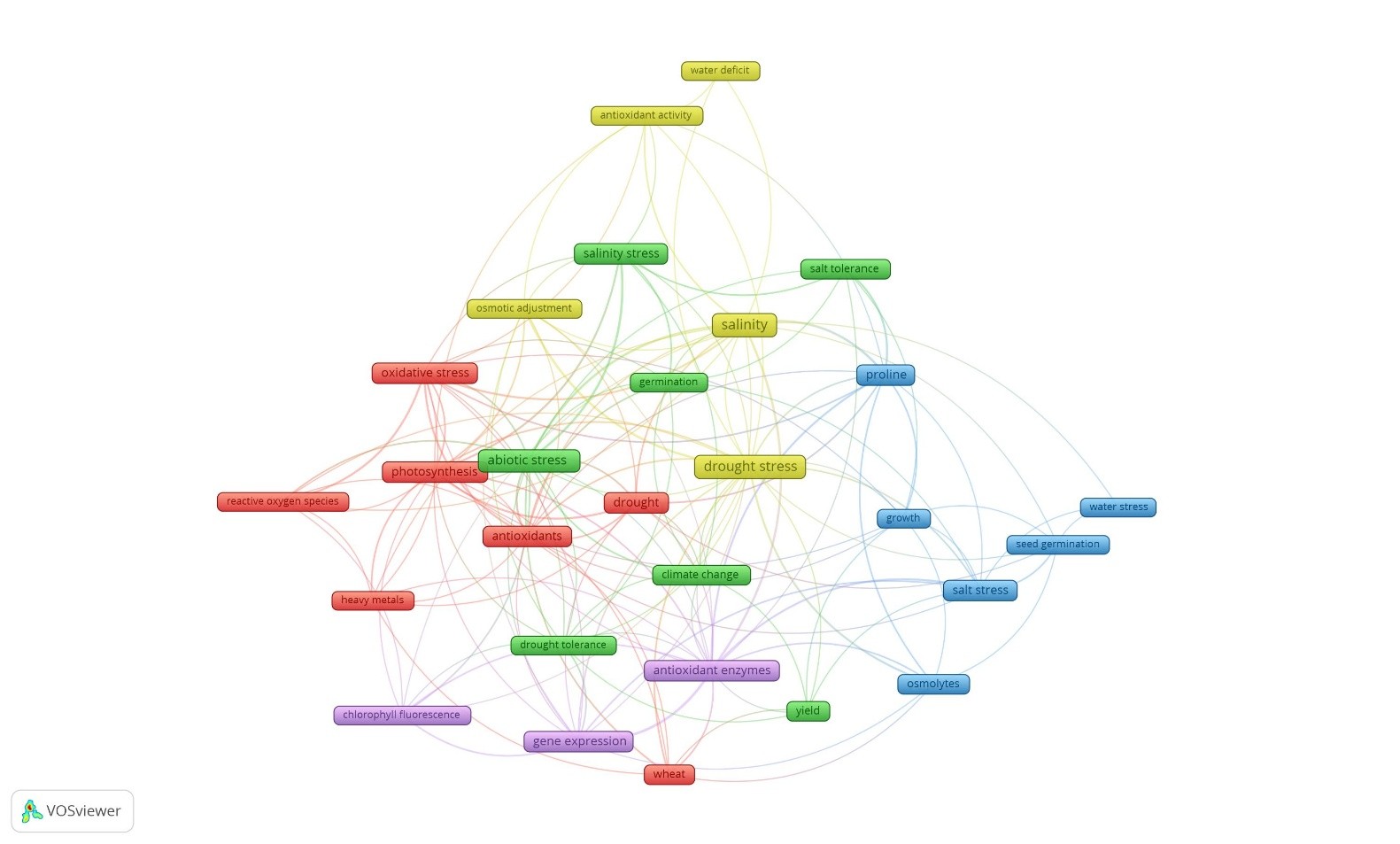

Supplement: Supplementary file 1 [file Image1.jpeg]
